# Management of Suspected Bladder Injury and Capsular Perforation After Holmium Laser Enucleation of the Prostate

**DOI:** 10.1089/cren.2018.0021

**Published:** 2018-06-01

**Authors:** Aye Lwin, Kieran Hynes, David Tzou, Joel Funk

**Affiliations:** ^1^ Department of Surgery, University of Arizona, Tucson, Arizona.; ^2^ Division of Urology, University of Arizona, Arizona Health Sciences Center, Tucson, Arizona.; ^3^ Department of Urology, University of California San Francisco, San Francisco, California.

**Keywords:** HoLEP, capsular perforation, BPH

## Abstract

***Background:*** Holmium laser enucleation of the prostate (HoLEP) is an attractive and well-studied alternative to transurethral resection of the prostate and open prostatectomy for the treatment of benign prostatic hyperplasia. There remains an established steep learning curve with relatively few complications described in the literature. A unique risk of HoLEP is injury of the bladder during morcellation of the adenoma and potential iatrogenic intraperitoneal bladder rupture. We present a rare complication of HoLEP demonstrated by two patients in which capsular perforation resulted in subsequent abdominal distention secondary to a large amount of irrigation fluid that leaked into the extraperitoneal space. Uniquely, these cases were managed differently, and serve as guidance to the HoLEP practitioner in postoperative management.

***Case Presentations:*** The first case involved a 74-year-old male who was found to have significant abdominal distention at the end of the procedure. Given an acute change in stability and concern for bladder injury during morcellation, a minilaparotomy was performed only to reveal extraperitoneal extravasation without intraperitoneal bladder injury or perforation. In the second case, a 78-year-old male undergoing HoLEP had a similar presentation of significant abdominal distention at the conclusion of morcellation. Given a low suspicion for any bladder injury, the patient was managed conservatively with diuretics. He was subsequently discharged on postoperative day 1.

***Conclusion:*** Capsular perforation is not a rare phenomenon that occurs during HoLEP. Rarely, perforations can lead to extravasation of irrigation fluid into the extraperitoneal space masquerading as a potential bladder injury related to morcellation because of the associated abdominal distention. This presentation can occur in large glands or early in a surgeon's learning curve when operative times are longer. When there is clear evidence to suggest there is no bladder injury, these cases can be managed conservatively and avoid the morbidity of an abdominal exploration.

## Introduction and Background

In recent years, holmium laser enucleation of the prostate (HoLEP) has emerged as an attractive alternative to transurethral resection of prostate (TURP) for the management of bladder outlet obstruction because of benign prostatic hyperplasia (BPH). HoLEP involves complete enucleation of the prostatic adenoma through laser dissection and subsequent removal of the prostatic tissue through morcellation in the bladder. This technique carries many advantages when compared with TURP and open prostatectomy, including effectiveness in the setting of massive (>100 g) glands, greater postoperative Qmax, improved reduction of subjective symptom scores, shortened catheterization times and hospital stay times, and decreased postoperative complication rates.^1^ Perioperative complication rates are low with the most common cited complications including capsular perforation, superficial bladder mucosal injury, ureteral orifice injury, and morcellator malfunction.^2^ To date, there has been one reported case of acute abdominal compartment syndrome involving massive fluid leakage into the retroperitoneal space during a HoLEP.^3^ We present two cases of intraoperative extraperitoneal fluid leakage during HoLEP.

## Case Presentations

### Case presentation 1

Patient 1 is a 74-year-old male who underwent HoLEP for refractory bladder outlet obstruction and bladder stones. His medical history included BPH complicated by recurrent urinary tract infections and bladder stones, elevated prostate specific antigen (biopsy negative), hyperlipidemia, and hypertension. Preoperative transrectal ultrasound (TRUS) estimated prostate volume to be 150 cc. HoLEP was performed utilizing a two-incision technique. Owing to the large amount of adenoma, extended time was spent during morcellation (120 minutes) because of poor observation secondary to bladder neck bleeding. There was noted to be an area of capsular perforation at the 5 o'clock position in the mid gland. When the operative drapes were removed, significant abdominal distention was noted.

In discussion with anesthetist, the patient's airway pressures upon induction ranged from 10 to 20, however, at this point in the procedure, the airway pressures had increased >30. The patient was also experiencing systolic pressures ranging from 80 to 90, whereas preoperatively he was >110 systolic. The drapes were removed at this time and the abdomen appeared distended and was firm on examination. Given the significant abdominal distention and concern for a bladder injury secondary to poor observation during morcellation, general surgery was consulted intraoperatively. Per the recommendation of general surgery, they elected to proceed with a subumbilical minilaparotomy, after initial laparoscopy was unsuccessful because of increased opening pressures with the Veress needle. Less than 400 cc of bloody-colored fluid was suctioned out of the abdomen, and an intraoperative cystogram was performed that revealed retroperitoneal extravasation without intraperitoneal bladder injury or perforation. As there was not a significant amount of intraperitoneal fluid, it was theorized that the capsular perforation resulted in extraperitonealization of intraoperative saline. Thus, a 10F Jackson Pratt drain was placed in the pelvis, and the fascia and skin were closed. He was given 20 mg of IV Lasix intraoperatively.

The patient was extubated effectively and transferred postoperatively to the ICU for hemodynamic monitoring and observation. In the ICU, cardiology was consulted because of a prolonged PR interval and bradycardia that ultimately warranted no further work-up. On postoperative day (POD) 1, the patient was progressing well, and he was transferred to the floor in stable condition. The Jackson-Pratt drain output was 710 cc on POD 0, 81 cc on POD 1, and then removed on POD 2 after draining 20 cc. The patient was discharged on POD 3. His catheter was removed on POD 10. Pathologic analysis of the specimen revealed no evidence of malignancy and 167 g specimen.

### Case presentation 2

Patient 2 is a 78-year-old male who was experiencing persistent lower urinary tract symptoms despite combined medical therapy with alpha blockade and 5-alpha reductase inhibitors. His medical history included coronary artery disease status post-coronary artery bypass grafting and percutaneous coronary intervention, and a history of pneumonia. Preoperative cystoscopy revealed enlarged median and lateral lobes, as well as severe trabeculations of the bladder with a TRUS measuring a 41 cc prostate. HoLEP was carried out utilizing a two-incision technique. Upon completion of morcellation, it was noted that the patient's abdomen was distended, but his peak airway pressures were normal, the abdomen was soft, and the catheter drainage was noted to be clear. In addition, there was no suspicion for a significant mismatch between irrigation used and fluid output collected in the drainage system.

**FIG. 1.**
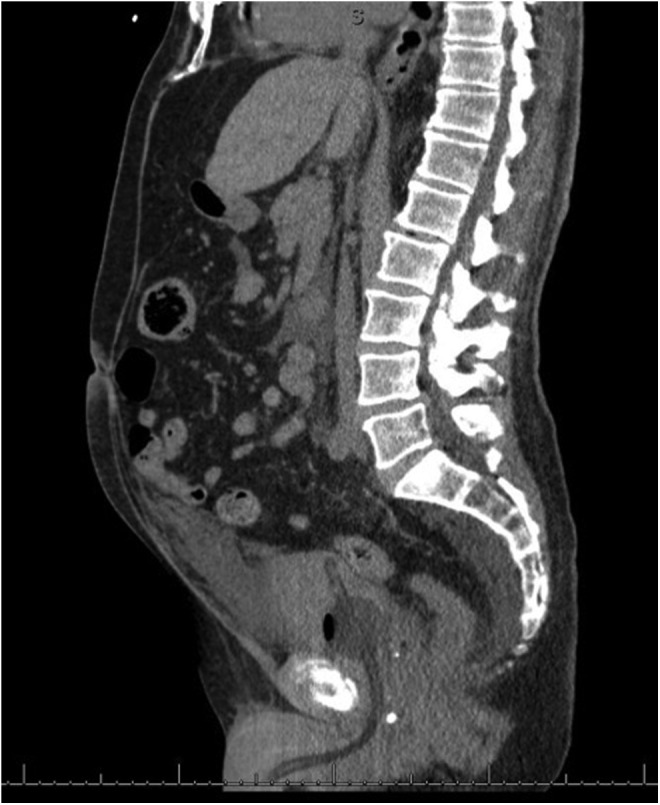
CT abdomen pelvis revealing a moderate amount of free fluid in the pelvis and upper abdomen. The fluid in the pelvis and lower abdomen is distributed in the extraperitoneal region.

**FIG. 2.**
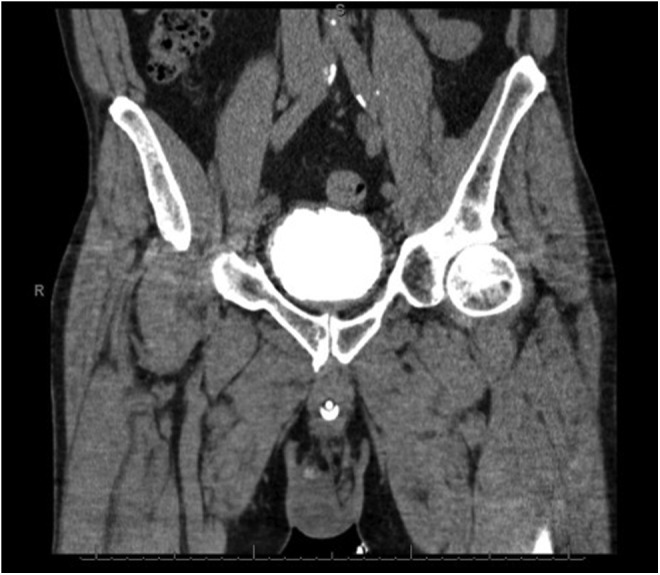
CT cystogram reveals no evidence of leak. Previous perivesicular fluid has resolved.

Given the previous similar presentation in Case 1 with no suspicion of bladder injury, we suspected that the patient had extraperitoneal extravasation of the saline irrigation through a capsular perforation as occurred in Case 1. The decision was made for the patient to be awakened, extubated, and transferred to the recovery room where he was further monitored. A stat noncontrast abdominal CT scan was performed that revealed a moderate amount of free fluid in the pelvis and upper abdomen; the fluid in the pelvis and lower abdomen was distributed in the extraperitoneal region with no evidence of hematoma (Fig. 1). The patient remained hemodynamically stable and was transferred to the floor with continuous bladder irrigation. The patient was given a 40 mg dose of Lasix ∼8 hours after the operation was completed. Overnight, there were no acute events. On POD 1, the patient's abdomen was soft and significantly less distended. The Foley catheter drained 3950 cc of urine overnight without evidence of hematuria. The patient was discharged with a catheter on POD 1. The patient had his catheter removed on POD 9. A postoperative CT cystogram revealed no evidence of leak with resolution of the pelvic and perivesical fluid (Fig. 2). Thirty grams of benign prostate tissue was removed on final pathology analysis. The patient was noted to have a bladder neck contracture seen on cystoscopy 4 months after his procedure for which he underwent cystourethroscopy and laser incision of bladder neck contracture.

## Discussion

Although HoLEP has emerged as a standard treatment of BPH, there remains a well-established steep learning curve associated with the operation.^2^ The most common cited complications include capsular perforation, superficial bladder mucosal injury, ureteral orifice injury, and morcellator malfunction.^2^ Capsular perforation rates have been documented from 0.3% to 10% and can potentially pose as a serious complication of HoLEP.^2^ This report increases the urologist's ability to recognize and conservatively manage a capsular perforation, resulting in a distended abdomen. Potential risk factors include cases with larger glands (>100 cc), prostates with thin capsules, or early surgeon's experience with longer operative times and suboptimal observation.

Capsular perforation can be characterized as threatened perforation, covered perforation, free perforation, or subtrigonal perforation.^2,4^ Threatened perforation is described as an area of diverging capsular fibers through which periprostatic fat might be visible. A covered perforation is similar to a threatened perforation but differs in degree with fatty tissue freely visible at the site of perforation, however, firmly covering the perforated hole. A free perforation is a perforation in the capsule through which irrigation fluid can be seen to run in and out, with little to no periprostatic fat observed at the margins of the wound. In a subtrigonal perforation, a false passage is created during insertion of the resectoscope or catheter, penetrating through the prostate and prostatic capsule into the direction of the trigone. This is likely to occur in large prostates especially with large median lobes. Threatened and covered perforations do not change the postoperative course of the patient, whereas free or subtrigonal perforations may lead to significant extravasation of irrigation fluid into the extraperitoneal space.^2^

Bladder injury during morcellation is another potential perioperative complication of HoLEP, with injury rates reported in 0.5% to 18.2% of cases.^1,2^ If unrecognized, large full-thickness injuries can allow extravasation of irrigation fluid into the extraperitoneal or intraperitoneal space. The extravasation of irrigation fluid can mimic the presentation of a free capsular perforation with fluid extravasation. Although it has not been reported, theoretically there is also the risk of bowel injury with a full thickness injury to the bladder. If there is suspicion for bladder injury, the bladder should be examined. Any injuries should either be repaired or managed conservatively with an indwelling catheter. Bladder injuries can be avoided by achieving optimal hemostasis and bladder distention before starting morcellation. Morcellation should take place away from the bladder mucosa.

As these cases demonstrate, a capsular perforation can mimic the presentation of a bladder injury during morcellation. If there is any suspicion of capsular perforation or bladder injury, an abdominal examination should be performed intraoperatively. Peak airway pressures may be increased or there may be a mismatch in irrigant inflow and outflow. With high suspicion of injury, both cystoscopic inspection of the bladder urothelium and additional imaging such as a cystogram or CT cystogram are indicated. If recognized correctly, the patient can be spared the morbidity of an exploratory laparotomy or laparoscopy and instead be treated conservatively with Lasix and bladder drainage through a urethral catheter. Patients who are clinically asymptomatic with normal vital signs can be discharged, generally on POD 1. Repeat imaging to ensure there is no bladder injury can be obtained on an outpatient basis.

## Conclusion

A rare complication of HoLEP is prostatic capsular perforation leading to large extravasation of irrigation fluid into the retroperitoneum, resulting in a distended abdomen. This presentation may be similar to that of a bladder injury, however, if there is low suspicion of a bladder injury, conservative measures can be taken for effective treatment.

## Abbreviations Used

BPH: benign prostatic hyperplasia
CBI: continuous bladder irrigation
CT: computed tomography
HoLEP: holmium laser enucleation of the prostate
POD: postoperative day
TRUS: transrectal ultrasound
TURP: transurethral resection of prostate
